# Fundus autofluorescence imaging using red excitation light

**DOI:** 10.1038/s41598-023-36217-x

**Published:** 2023-06-19

**Authors:** Johannes Birtel, Tobias Bauer, Laurenz Pauleikhoff, Theodor Rüber, Martin Gliem, Peter Charbel Issa

**Affiliations:** 1 Oxford Eye Hospital, John Radcliffe Hospital, Oxford University Hospitals NHS Foundation Trust, Oxford, OX3 9DU UK; 2 Nuffield Laboratory of Ophthalmology, Nuffield Department of Clinical Neurosciences, University of Oxford, Oxford, UK; 3 Department of Ophthalmology, University Medical Center Hamburg-Eppendorf, Hamburg, Germany; 4 Department of Epileptology, University Hospital Bonn, Bonn, Germany

**Keywords:** Retinal diseases, Eye diseases

## Abstract

Retinal disease accounts significantly for visual impairment and blindness. An important role in the pathophysiology of retinal disease and aging is attributed to lipofuscin, a complex of fluorescent metabolites. Fundus autofluorescence (AF) imaging allows non-invasive mapping of lipofuscin and is a key technology to diagnose and monitor retinal disease. However, currently used short-wavelength (SW) excitation light has several limitations, including glare and discomfort during image acquisition, reduced image quality in case of lens opacities, limited visualization of the central retina, and potential retinal light toxicity. Here, we establish a novel imaging modality which uses red excitation light (R-AF) and overcomes these drawbacks. R-AF images are high-quality, high-contrast fundus images and image interpretation may build on clinical experience due to similar appearance of pathology as on SW-AF images. Additionally, R-AF images may uncover disease features that previously remained undetected. The R-AF signal increases with higher abundance of lipofuscin and does not depend on photopigment bleaching or on the amount of macular pigment. Improved patient comfort, limited effect of cataract on image quality, and lack of safety concerns qualify R-AF for routine clinical monitoring, e.g. for patients with age-related macular degeneration, Stargardt disease, or for quantitative analysis of AF signal intensity.

## Introduction

Fundus autofluorescence (AF) imaging visualizes fluorophores at the posterior pole. It is a standard method for characterizing, diagnosing, and monitoring retinal disease and is an established outcome measure in clinical trials. The currently most widely used fundus AF imaging devices use short-wavelength (SW) excitation light in the blue or green range. The main fluorophore at this wavelength is lipofuscin which accumulates with ageing in the retinal pigment epithelium (RPE)^1–4^. Its increased or decreased abundancy is considered to play a role in the pathophysiology of various degenerative retinal diseases^5^.

SW-AF imaging has several limitations, which include (I) glare and discomfort during image acquisition^6,7^; (II) reduced image quality and AF signal intensity in the elderly due to cataract^5,8^; (III) limited visualization of the central retina due to absorption of the blue excitation light by macular pigment^3,5,9^; and (IV) potential retinal light toxicity, particularly in patients with inherited retinal diseases^10–16^.

The limitations of SW-AF do not apply or should be less relevant when longer wavelength (LW) excitation light is used. However, the currently available near-infrared AF (785 nm excitation, NIR-AF) is characterized by poor AF signal intensity with reduced and less reliable image quality^17–19^. LW excitation with wavelengths shorter than 785 nm should have a higher efficiency^20,21^.

To leverage the potential of LW-AF, we equipped a custom-built confocal scanning laser ophthalmoscope (cSLO) with red excitation lasers (642 nm and 705 nm; R-AF). This study aimed to evaluate the feasibility, performance and potential advantages of this new imaging modality. Moreover, we provide first evidence that R-AF imaging may have added value when assessing patients with cataract and various retinal diseases, including age-related macular degeneration (AMD) and Stargardt disease.

## Results

**Figure 1:**
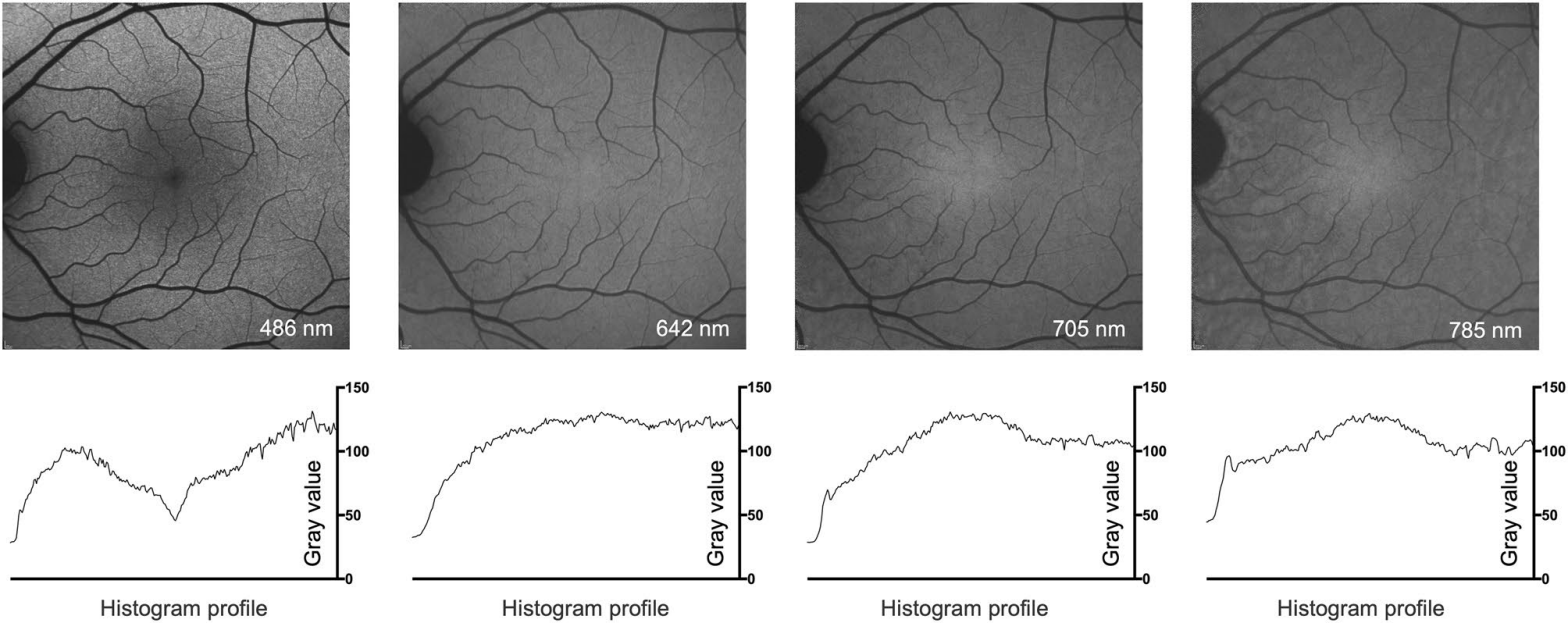
Blue-, red- and near-infrared excitation fundus autofluorescence imaging in the normal retina. The upper row displays unprocessed fundus autofluorescence (AF) images in a healthy subject using short-wavelength (486 nm), red (642 and 705 nm) and near-infrared (785 nm) excitation light. The lower row illustrates the horizontal grayscale histogram profile (horizontal line through the fovea, 1379 pixels wide) of the image above. The AF signal intensity is displayed in an 8-bit grayscale image (range, 0–255). Low pixel values (dark) indicate low AF intensities and high pixel values (light) represent high AF intensities.

In normal eyes, R-AF images using 642 nm and 705 nm excitation light showed a consistent pattern. The AF intensity increased slightly from the temporal optic disc margin towards the fovea. In the foveal region (approximately central 5°), signal intensity was usually similar to more eccentric areas using 642 nm excitation light, but was slightly increased with 705 nm excitation light, similar to the usual appearance on 785 nm-excited AF images (Fig. 1).

The potential for detailed assessment of the fovea, the central retinal area that enables high-resolution vision, was particularly evident in comparison to images acquired with 486 nm excitation light, where the macular AF is reduced by the absorption of SW-light by macular pigment and melanin. The RPE-derived AF signal intensity with 785 nm excitation light was lower overall when compared with the other 3 modalities, resulting in poorer contrast. In accordance with the absorption spectrum of hemoglobin^22^, retinal vessels showed a lower AF signal (relative to background autofluorescence) at 486 nm excitation light, compared to 642 nm and 705 nm excitation light (Supplemental Results 1 and 2).

### Image acquisition and analysis

To test whether AF signal intensity changes with exposure to the excitation light, as observed due to bleaching of photopigment when using blue excitation light^23^, eyes were imaged over 30 s, and grey levels were analyzed over time. No relevant change of the R-AF signal was observed at the fovea or at 12°–17° eccentricity (Supplemental Results 3).

Next, we assessed the effect of defocus on measured AF signal intensity. A slight defocus, which may occur in routine imaging, had no marked impact on the AF signal intensity; a deviation from the focal plane of ± 0.42 D (642 nm) and ± 0.30 D (705 nm), respectively, resulted only in a 5% decrease of the AF signal intensity (Supplemental Results 4).

A linear relation between AF signal intensity and laser power was observed when the laser power was reduced stepwise from its maximum (arbitrary set 100) to 25 with no change of the detector settings (Supplemental Results 5). This assessment also allowed us to estimate the required 642 nm and 705 nm laser power that may result in an AF signal comparable to that using 486 nm excitation light with laser power currently used in clinical routine imaging (Supplemental Results 6). With these fixed laser powers for R-AF, grey levels were determined by the detector settings and followed the equation of

$$GV_{signal} (88) = GV_{signal} (D) \cdot 1.0947^{(88 - D)}.$$

Here, *GV*_*signal*_ is the measured grey value, *D* is the detector setting used for recording of the image, and 88 is an arbitrarily chosen detector setting that usually results in mid-range grey levels when the standard 486 nm AF imaging is performed in a middle-aged adult without retinal disease. This equation provides arbitrary units (AU) of the AF signal intensity and facilitates quantitative analysis with different detector settings (for more details see Supplemental Results 7).

To investigate test–retest repeatability, subjects moved away from the instrument, the focus was randomly changed while the sensitivity and laser power settings remained fixed. This revealed a repeatability of ± 12% (642 nm) and ± 9% (705 nm), respectively. This measure indicates that higher variation between 2 measurements would only occur in 5% of occasions (95% confidence interval). For additional repeatability and quantitative measures see Supplemental Results 8.

### Clinical application: glare, cataract, quantification of autofluorescence intensity

**Figure 2:**
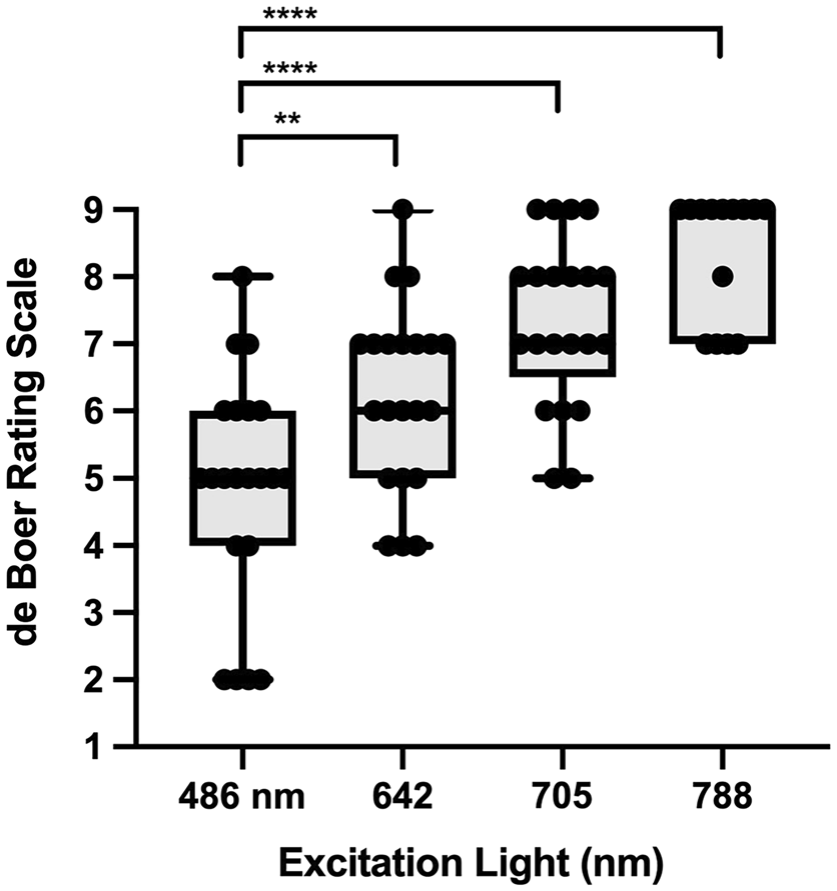
Perceived glare of different excitation wavelengths. Discomfort glare (n = 21) of four different excitation wavelengths (486 nm, 642 nm, 705 nm, 788 nm) during retinal imaging (10 s). The magnitude of discomfort glare was evaluated using the de Boer rating scale which uses a 9-point response scale according to descriptors attached to odd-numbered points (1 = Unbearable, 3 = Disturbing, 5 = Just acceptable, 7 = Satisfactory, 9 = Just noticeable). For this assessment, laser power settings resulting in equivalent autofluorescence intensities were used (see Supplemental Fig. 6). **** p < 0.0001, ** p = 0.006.

To compare perceived glare of different excitation wavelengths, patients and healthy individuals were asked to rate glare during imaging (10 s) using the subjective de Boer scale which describes the magnitude of discomfort using a 9-point response scale^24^.  Discomfort glare depended on excitation light (Kruskal–Wallis test; p < 0.0001). Image acquisition using 486 nm excitation light was rated significantly more uncomfortable (median: 5.0; interquartile range [IQR] 4–6) compared to 642 nm (median: 6; IQR 5–7), 705 nm (median: 7; IQR 6.5–9), and 788 nm (median: 9; IQR 7–9) (Fig. 2).

**Figure 3:**
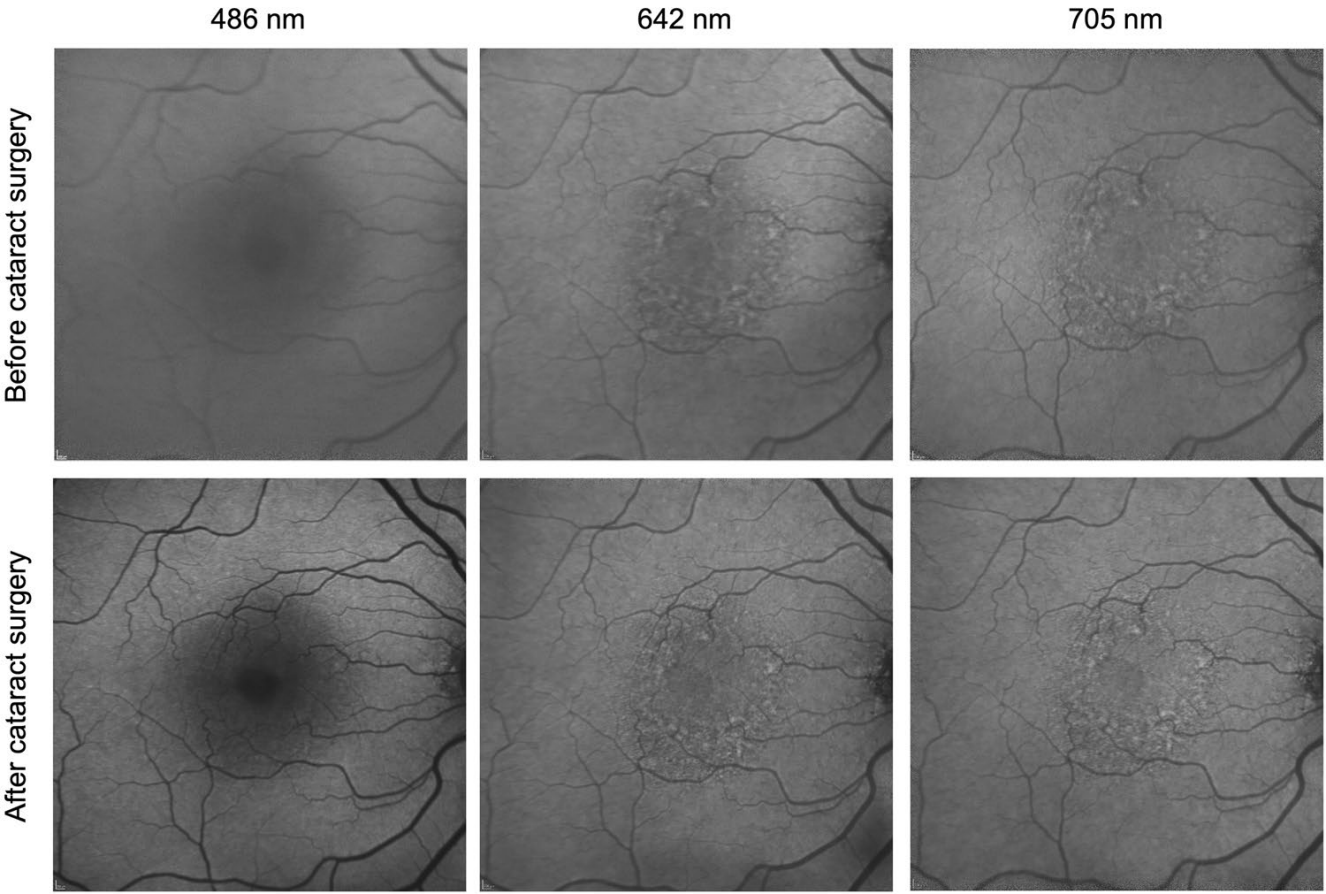
Impact of cataract on image quality using blue- and red-excitation fundus autofluorescence (AF) imaging. Fundus AF imaging with short-wavelength (486 nm) and red excitation light (642 nm, 705 nm) in a patient before (upper row) and after (lower row) cataract surgery. The nuclear cataract in this patient had no substantial effect on the image quality using 642 nm and 705 nm excitation light, whereas the image quality and subsequently the evaluation of the retinal morphology was considerably reduced with 486 nm excitation light. All images are unprocessed recordings.

In patients with cataract, image quality and evaluation of the retinal morphology is considerably reduced on images acquired with 486 nm excitation light. However, the example of a patient imaged before and after cataract surgery shows that cataract has comparatively much lesser impact on the image quality when 642 nm or 705 nm excitation light is used (Fig. 3).

The potential of R-AF for quantitative analysis of lipofuscin-associated AF is demonstrated in a patient with *ABCA4*-related retinopathy (Stargardt disease). Compared to healthy controls, the patient showed an increased AF intensity using 642 nm excitation light, comparable to measurements using SW-AF. When using 705 nm excitation light, the increase was less pronounced (Supplemental Results 9).

### Case studies

In the following, some clinical examples are presented to illustrate the clinical value of R-AF imaging.

**Figure 4:**
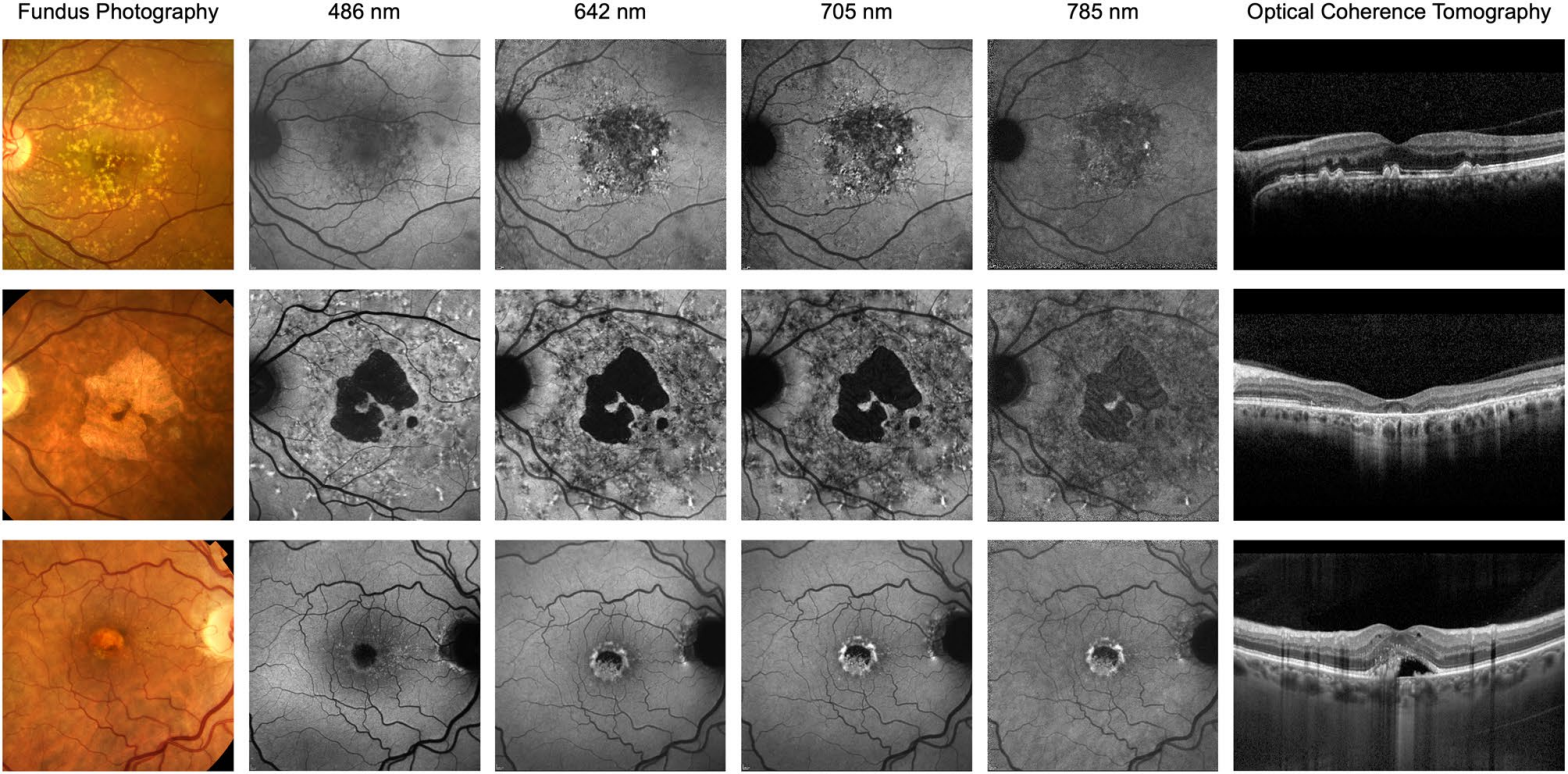
Multimodal imaging including red-excitation fundus autofluorescence (AF) imaging. Color fundus photograph, fundus AF images using short-wavelength (488 nm), red (642 nm and 705 nm) and near-infrared (785 nm) excitation light, and optical coherence tomography images through the foveal centre (top and middle rows: horizontal, bottom row: vertical). Top row: Age-related macular degeneration. Middle row: Late-onset Stargardt disease. Bottom row: Vitelliform macular lesion. All AF recordings are normalized for optimal contrast.

The first example (Fig. 4, top row) shows images of a patient with macular drusen due to age-related macular degeneration. While SW-AF shows mild alterations limited to the central retina (approximately 10°), R-AF shows substantial loss of the AF signal in the same area and more eccentric focal changes. The severe macular changes identified on R-AF imaging showed no obvious correlate on the OCT- and color fundus images.

The second case is a patient with Stargardt disease (Fig. 4, middle row). The R-AF images appear as a composite of the SW-AF and NIR-AF images: While the area of RPE atrophy is better defined on SW- and R-AF images, the latter also show adjacent areas of reduced AF, similar to those observed on the NIR-AF image. A detailed analysis of the foveal region is possible on R-AF images, which is limited on the SW-AF image due to the masking by macular pigment.

Such improved assessment of the fovea is also illustrated in a patient with a vitelliform macular lesion (Fig. 4, bottom row). The R-AF image shows a likely round loss of the RPE-derived AF signal and increased autofluorescence associated with subretinal material mainly in the inferior half of the lesion. These features are not readily visible on the SW-AF image.

**Figure 5:**
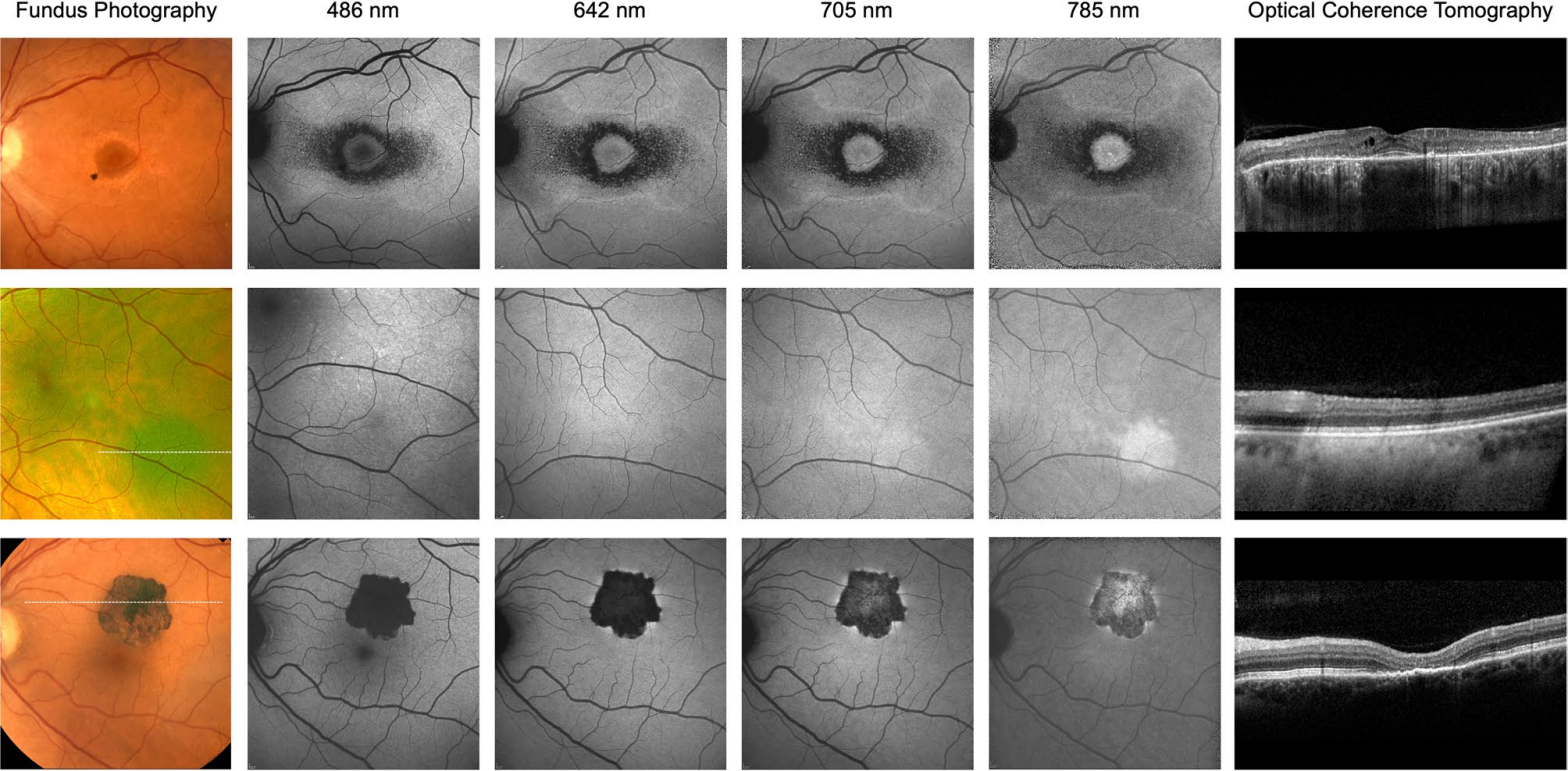
Multimodal imaging including red-excitation fundus autofluorescence (AF) imaging. Color fundus photograph, fundus AF images using short-wavelength (488 nm), red (642 nm and 705 nm) and near-infrared (785 nm) excitation light, and optical coherence tomography images (top: horizontal through the foveal center, middle and bottom rows: position as illustrated in the color image). Top row: Hydroxychloroquine retinopathy. Middle row: Choroidal naevus. Bottom row: Congenital hypertrophy of the retinal pigment epithelium. All AF recordings are normalized for optimal contrast.

R-AF images revealed similar findings as observed on SW-AF images in a case of hydroxychloroquine retinopathy (Fig. 5, top row) and for various retinal dystrophies (not shown). Pigmented fundus lesions usually show an increased signal on NIR-AF, but not on SW-AF images. Such pigmented lesions appeared similar to SW-AF when recorded with 642 nm excitation and similar to NIR-AF, but with fainter signal intensity, when 705 nm excitation light was used (Fig. 5, middle and bottom row).

## Discussion

Retinal imaging is a key technique to diagnose and monitor retinal disease. Here, we investigate a novel fundus AF technique using red excitation lasers (642 nm and 705 nm) built into a cSLO. We hypothesized that improved macular assessment would be possible with fewer safety concerns, improved patient comfort, and less influence by cataract compared to short-wavelength AF imaging.

Very little was previously known about the in vivo excitation efficiency of red light for fluorophores at the ocular fundus. In cryosections of human donor eyes, excitation with 633 nm light resulted in a higher AF signal from the RPE relative to Bruch’s membrane and subretinal deposits when compared to shorter excitation wavelengths (364 nm, 486 nm, and 568 nm)^25^. This indicated that lipofuscin distribution could be mapped with red excitation light, but the study methodology did not allow conclusions on whether the signal intensity would be sufficient for in vivo AF imaging. Our study illustrates the clinical applicability of fundus AF imaging using red excitation light. The topographic distribution of the AF signal with 642 nm excitation light is consistent with previous studies that mapped lipofuscin distribution, often with a localized foveal dip (not shown). In contrast, the slightly higher foveal signal with 705 nm excitation may indicate a more relevant contribution of melanin-/melanolipofuscin-derived AF with increasing wavelength of the excitation light, in line with the observation that the NIR-AF (785 nm) signal strongly depends on the presence of melanin.

Fundus AF imaging using short-wavelength excitation light is considered safe^26,27^. However, the rationale for safety is based on ANSI thresholds for the healthy retina, whereas ophthalmic imaging is typically performed in patients with retinal disease. Thus, published standards do not cover a potentially lower individual threshold for light damage, e.g. through mutation-specific effects, a reduced reserve for recovery, or the contribution of endogenous photosensitizers, particularly in patients with retinal dystrophies^19^. Light-dependent acceleration of retinal degeneration in susceptible retinas has, for instance, been shown in an animal model with rhodopsin mutation^12–14,16,28^. Replacing blue for red excitation light for fundus AF imaging results in lower energy per photon, and photochemical toxicity does not need to be considered for light with wavelengths > 600 nm. This allows to apply more laser energy and to compensate for potentially lower fluorescence efficiency. Moreover, retinal light damage is thought to be tightly linked to light absorption by rhodopsin, but red light is outside its absorption spectrum and tissue absorption at wavelengths between 600 to 1300 nm is relatively low^12–14,29^. Cones are much less vulnerable to light damage, but even if there is a role for cone opsins, absorption efficiency is low at 642 nm and 705 nm^30^.

Substitution of potentially harmful short-wavelength light by safer longer wavelength light appears particularly logical for repetitive examinations and for quantitative AF intensity measurements which may be used as a biomarker for lipofuscin accumulation^15,16^. Besides its diagnostic usefulness, such quantitative R-AF imaging may serve as a safe outcome measure in clinical trials that aim at lowering lipofuscin accumulation. Our study provides first evidence that R-AF may indeed be used to quantify lipofuscin-associated AF intensity, without the requirement of photopigment bleaching.

Retinal imaging using visible light is often associated with considerable discomfort glare^31,32^. Photophobia increases with decreasing wavelength, and it was therefore expected that R-AF would be better tolerated^6,7^. However, discomfort glare also depends on the light intensity, and it was unknown if excitation light resulting in a clinically useful AF signal intensity would be more comfortable than short-wavelength excitation light^6,7^. In this study, we show that R-AF imaging with excitation light intensities resulting in similar AF signal as on conventional SW-AF is indeed associated with improved patient comfort.

Media opacities, in particular nuclear cataracts, have a relevant impact on image quality on SW-AF images, often preventing precise analysis of retinal changes^8^. Lenses with cataract absorb and scatter the blue excitation light, and their autofluorescence may reduce the contrast of the fundus AF image^5^. On the contrary, R-AF imaging in patients with cataract resulted in high-quality recordings and hence would be a more favorable imaging modality for patients with cataracts, even though it requires additional studies to investigate the exact impact of cataract types and severity.

Finally, this study provides first evidence of the clinical usefulness of R-AF imaging. The observed AF patterns were remarkably similar to findings on SW-AF imaging and hence, clinicians may build on previous experience for image interpretation. Moreover, we demonstrate that additional information can be embedded in R-AF images. For instance, R-AF in *ABCA4*-related retinopathy showed the combined information of SW-AF and NIR-AF alterations, hence allowing a more comprehensive analysis by means of only one image (Fig. 4). Of note, NIR-AF images are rarely recorded in clinical routine due to their lower contrast, but clinical information encoded in NIR-AF images of patients with *ABCA4*-related retinopathy may be functionally relevant and may uncover early pathology^33–37^. The observation that characteristic flecks in *ABCA4*-related retinopathy usually show a high signal on SW-AF but not on R-AF supports previous reports suggesting that these flecks do not represent lipofuscin accumulation in the RPE, but are rather composed of different fluorophores that may accumulate in the subretinal space^34,37^. This also illustrates that various fluorophores at the ocular fundus may only be visualized using specific excitation wavelengths and that such differences may inform on deviating pathophysiological processes. Another example for additional information on R-AF images is the loss of AF signal in a central retinal area of an eye with AMD that shows no correlate on fundus color photography, SW-AF- or OCT-imaging (Fig. 4). Understanding the cause of this appearance as well as the significance with regards to disease stratification and prognostication will require further studies. Furthermore, R-AF allows improved analysis of foveal pathologies because red light is not absorbed by macular pigment. The resulting lack of foveal masking, which limits foveal assessment when blue-excitation light is used, allows a detailed evaluation of macular diseases, including AMD and Stargardt disease^9,38,39^.

Limitations of R-AF include that red light is outside the excitation spectrum of fluorescein and hence cannot be used for fluorescein angiography. However, if R-AF imaging would be incorporated in future devices as an additional modality, it could be performed between mid- and late-phase fluorescein angiography recordings and hence improve clinic flow. Red light may also be inefficient for exciting indocyanine green, but the feasibility of ICG angiography would require further investigations^40^. As red light is not absorbed by macular pigment (lutein and zeaxanthin) characteristic lack or redistribution of macular pigment as observed e.g. associated with foveal hypoplasia or in macular telangiectasia type 2 may not be detected^41,42^. Furthermore, we investigated only a relatively small number of subjects and diseases; hence, general conclusions will require further disease-specific studies.

In conclusion, R-AF allows comprehensive retinal analysis, may provide additional information about retinal disease, and may facilitate retinal imaging with improved patient comfort and fewer safety concerns compared to the currently most widely used AF technologies. Quantitative R-AF imaging may serve as a sensitive and safe outcome measure in clinical trials that aim at lowering lipofuscin accumulation. Thus, R-AF holds great potential for both clinical practice and (clinical) research and appears to be an ideal retinal imaging modality.

## Methods

### Participants

This prospective, cross-sectional study (NCT03592017, first registered 19/07/2018) was conducted at the Oxford Eye Hospital, Oxford University Hospitals NHS Foundation Trust, and the Nuffield Laboratory of Ophthalmology, Nuffield Department of Clinical Neurosciences, University of Oxford, Oxford, United Kingdom. The study was in adherence to the declaration of Helsinki. Institutional review board approval (Health Research Authority, South Central—Oxford C Research Ethics Committee) and patients' informed consent were obtained.

In total, 80 participants were investigated, including 15 healthy subjects with normal retinal status, good fixation, and clear media and 65 patients with retinal disease.

### Safety considerations

Following the recommendations of the American National Standards Institute (ANSI), the retinal light exposure of the R-AF imaging lasers is markedly (≈ 8–12 times) below the accessible emission limit (AEL) of class 1 laser products^26,27^. As the wavelength of both R-AF lasers is > 600 nm, no photochemical retinal hazard has to be considered; photochemical retinal hazard is a fundamental element in the safety review of SW-AF (see the Supplementary Methods for additional information).

### Image acquisition

We used a custom-built confocal scanning laser ophthalmoscope (Heidelberg Engineering, Heidelberg, Germany) equipped with lasers for excitation at 642 nm (barrier filter: 652 nm), 705 nm (barrier filter: 715 nm), and 785 nm (barrier filter: 798 nm). The study focused on investigating the performance of red-light fundus autofluorescence (R-AF) using the 642 nm and 705 nm excitation lasers. For selected clinical aspects, images were compared with NIR- and SW-AF images. The latter were recorded using the 486 nm laser (barrier filter: 496 nm) of a commercial Spectralis HRA + OCT (Heidelberg Engineering) (see Supplemental Methods 1 for additional information).

Before imaging, pupils were dilated by instillation of 0.5% tropicamide and 2.5% phenylephrine. The camera was positioned centered on the fovea of the participant by using the near-infrared reflectance mode and the internal fixation light. After switching to the AF mode, focus and alignment were readjusted to obtain a maximum and uniform signal. The optimal camera position was re-checked, and the participant was asked to blink a few times to provide optimal imaging conditions. A series of 12 successive images were recorded (30° field of view and 1536 × 1536 pixels) and a quality check was performed after the recording of each series. In case of insufficient quality (e.g. due to inhomogeneous illumination, unstable fixation, sectorial opacities such as eyelashes or floaters), images were excluded from further analysis.

Recorded AF images display the spatial distribution of the AF signal intensity for each pixel in an 8-bit grayscale image (range, 0 to 255). Low pixel values (dark) indicate low AF intensities and high pixel values (light) represent high AF intensities^5^. Unless stated otherwise, no image processing was performed. Histogram stretching (“normalization”) was used to improve contrast in cases where only qualitative assessment was performed.

### Image analysis

An in-house image processing pipeline, written in Python and based on the open-source SciPy library, was used for image analysis. First, the foveal center and the temporal neuroretinal edge of the optic disc were manually labeled and checked by an additional rater. The distance between the edge of the disc and the foveal center (FD) was then used to align and scale the measurement areas which was adapted from previous reports^4,23,43–46^. Measurements were performed in four concentric rings around the fovea and a circular foveal segment; the three outer rings were divided into eight segments and the innermost ring was divided into four segments (Supplemental Methods 2). The image processing pipeline accounted for the presence of vessels by fitting the sum of two Gaussians to the histogram of every segment as performed in previous studies^4,23,43–45^. Here, we assume that the smaller Gaussian accounts for lower grey values of the vessels and the larger Gaussian for the grey values of the fundus background. Thus, the minimum grey value of the fundus is then set at the 5%-quantile of the larger Gaussian. Segments extending beyond 15° from the center of the image were excluded. The grey values were corrected by subtracting the “Grey value offset” which is the signal measured in the dark mode (detector on, laser modulated off). The respective values of each image are provided by the Heidelberg Spectralis software^46^.

### Statistical analyses

Statistical Analyses were performed using SciPy, Microsoft Excel (Microsoft, Redmond, WA, USA), and graphing software (GraphPad Prism, La Jolla, CA, USA). To measure test–retest repeatability and agreement between two measures obtained from different image acquisition modes, the method of Bland and Altmann was used^47^. The coefficient of repeatability (or agreement) between two measures qAF_1_ and qAF_2_, expressed in percent, is repeatability = ± 1.96 ⋅ σ_qAF_ ⋅ 100, where σ_qAF_ is the standard deviation of Δ_qAF_ = 2(qAF_1_ − qAF_2_)/(qAF_1_ + qAF_2_) across all considered image pairs. The here presented repeatability coefficient can be interpreted as a 95% confidence interval for testing under the same conditions, while the analogously defined agreement coefficient can be interpreted as a 95% confidence interval for switching between different image acquisition modes.

## Supplementary Information

Supplementary Information.

## Data Availability

The datasets generated and analyzed during the current study are not publicly available due to data protection regulations but are available from the corresponding author on reasonable request.
