# Supplementary material for: Fundus autofluorescence imaging using red excitation light
Source: Sci Rep. 2023 Jun 19;13:9916. doi: 10.1038/s41598-023-36217-x (PMC10279676; doi:10.1038/s41598-023-36217-x)
Supplement: Supplementary file 1 — Supplementary Information. [file 41598_2023_36217_MOESM1_ESM.pdf]

Fundus autofluorescence imaging using red excitation light

– Supplement –

Johannes Birtel, Tobias Bauer, Laurenz Pauleikhoff, Theodor Rüber,
Martin Gliem, Peter Charbel Issa

Supplemental Results 1: Histogram profiles comparing retinal vessels to background fundus autofluorescence

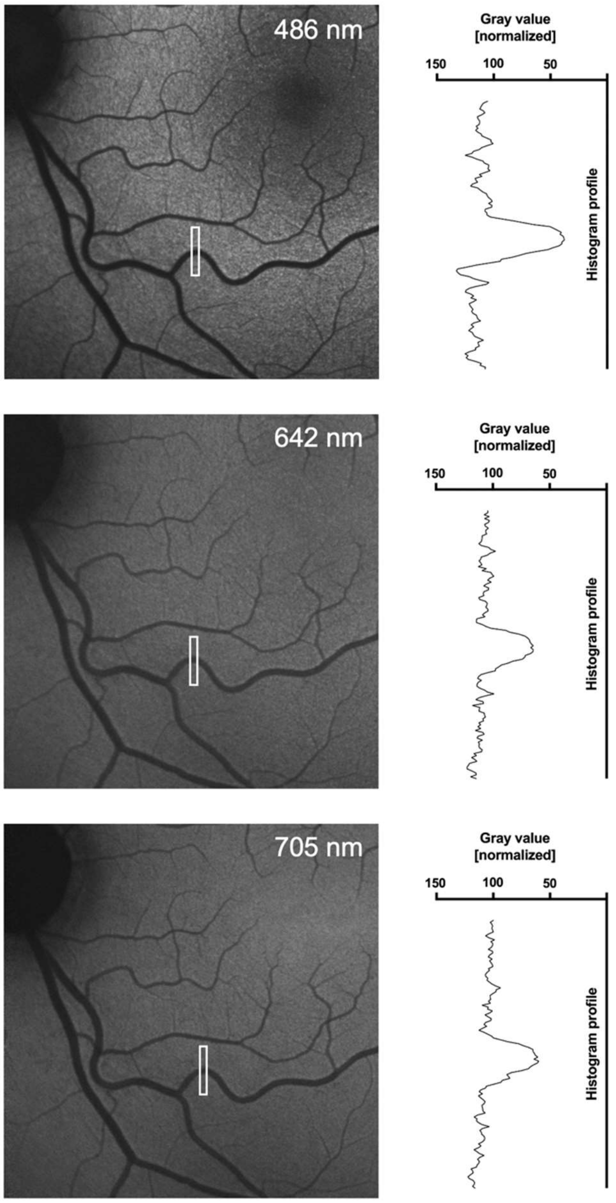

This figure contrasts grey levels of vessels to the background fundus autofluorescence (AF) by grayscale histogram profiles. Compared to the background AF, vessels exhibit a lower AF signal at an excitation wavelength of 486 nm than at 642 nm and 705 nm excitation. The white frame indicates the measurement areas for the histogram profile (length: 140 pixels, width: 15 pixels). Images with equivalent autofluorescence intensities in the central eight segments (see Supplemental Methods 2) were used for analysis. For normalization, the mean of the first 20 pixels (frame, starting from inferior) of the three wavelengths was determined. The mean of 486 nm was used as reference (factor 1), the grey values using 642 nm (factor 1.17) and 705 nm (factor 1.25) were normalized to this.

Supplemental Results 2: Fundus autofluorescence intensity at the optic nerve head and a vessel compared to background

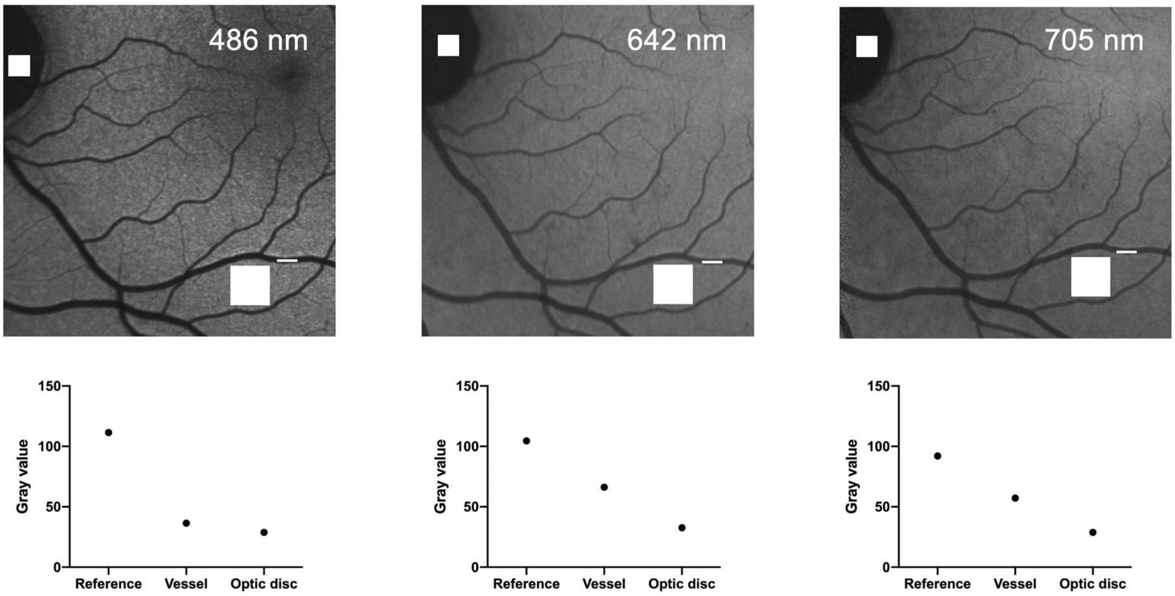

This figure contrasts grey levels representing fundus autofluorescence (AF) intensity of a vessel (linear small frame) and of the optic nerve head (small frame) to a background reference (large frame) at an excitation wavelength of 486 nm, 642 nm, and 705 nm. For all measurement areas the mean grey level of the area is displayed. At similar background AF intensity, AF levels at the optic disc are similar across the three excitation wavelengths. However, AF intensity measured over vessels differs and is relatively lower at 486 nm excitation compared to 642 nm or 705 nm excitation.

Supplemental Results 3: Effect of exposure to excitation light on autofluorescence intensity

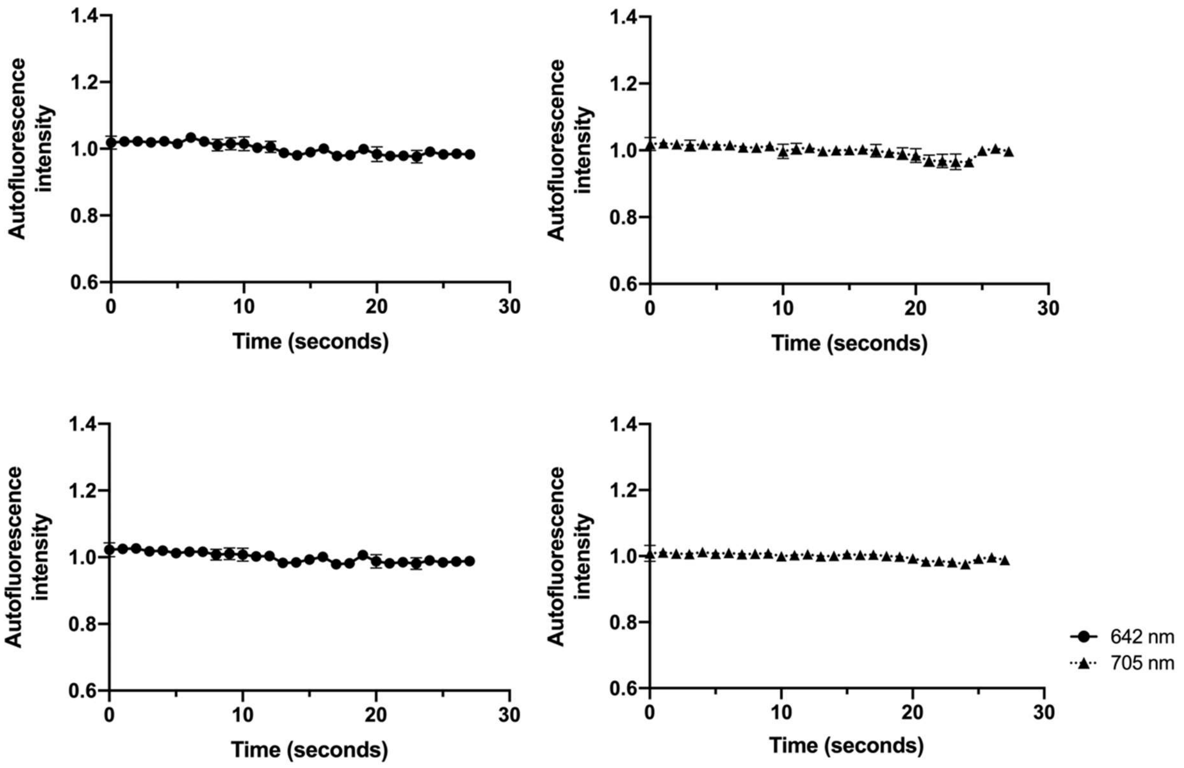

To test whether fundus autofluorescence (AF) signal intensity changes with exposure to the excitation light, 3 eyes were imaged over 30 seconds (4.7 images/second), and the autofluorescence intensity was analyzed over time (normalized to the mean of each 30 second series). Using 642 nm (left) and 705 nm (right) excitation light, no relevant change or fluctuation of the AF intensity was observed at the foveal region (top row) or at the central eight-segments (lower row), respectively (measured regions illustrated in Supplemental Methods 2). The error bars display the standard error of mean.

Supplemental Results 4: Change of autofluorescence intensity measures with defocus

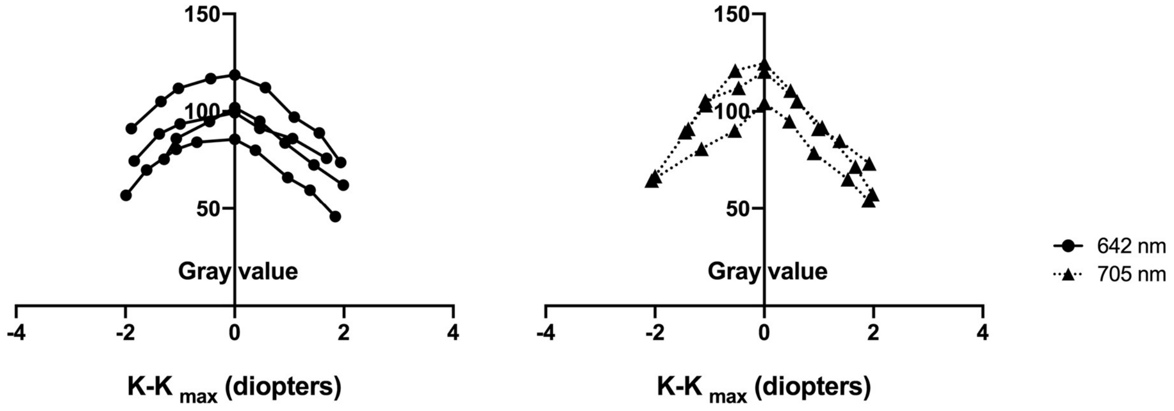

This graph illustrates the change of fundus autofluorescence (AF) intensity with a defocus of up to ± 2 D from the confocal plane of maximum AF signal intensity. Deviation of ±0.42 D (642 nm) and ±0.30 D (705 nm), respectively, resulted in a 5% decrease of grey levels (mean of central eight-segments). A ±0.5 D or ±1 D deviation from the focal plane resulted in a decrease of AF intensity of 5.9% or 12.6% (642 nm) and 8.3% or 20.4% (705 nm), respectively.

Supplemental Results 5: Impact of excitation light intensity on the autofluorescence signal

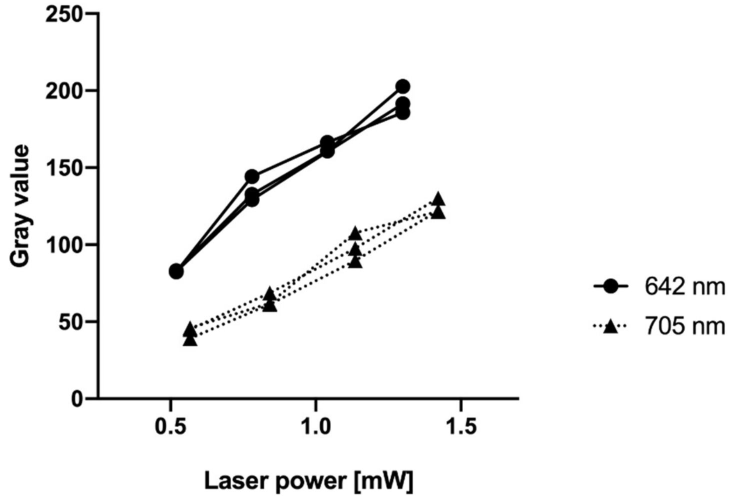

The impact of excitation light intensity on the fundus autofluorescence (AF) signal was investigated by reducing the laser power from its maximum (arbitrary unit [AU] of 100, equivalent to 1.30 mW at 642 nm; 1.42 mW at 705 nm) to AUs of 75 (1.04mW at 642 nm; 1.13 mW at 705 nm), 50 (0.78 W at 642 nm; 0.84 mW at 705 nm), and 25 (0.52 W at 642 nm; 0.56 mW at 705 nm).

Supplemental Results 6: Laser power for 486, 642 and 705 nm excitation light to obtain equivalent autofluorescence intensities

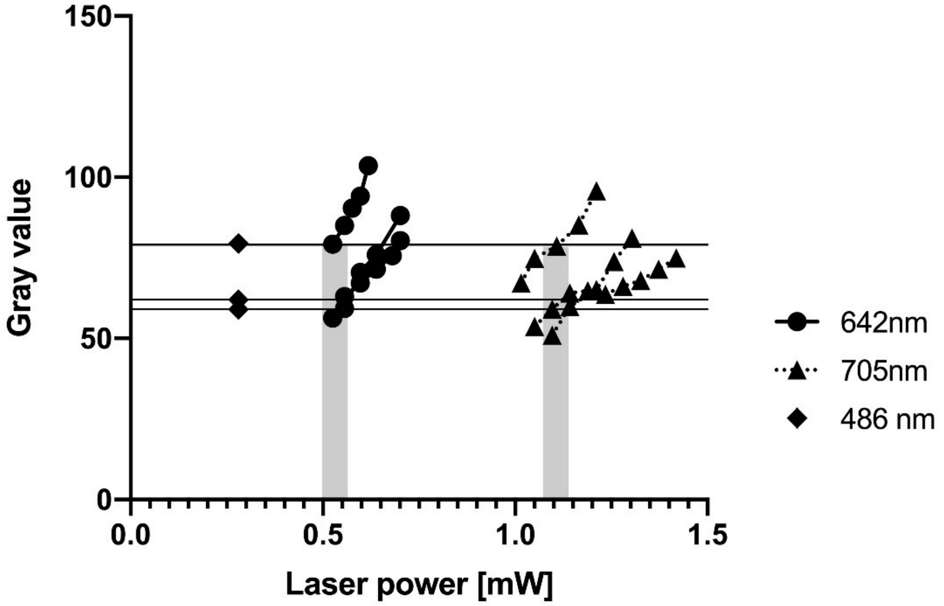

This graph illustrates the red excitation fundus autofluorescence (R-AF) laser power settings which result in a fundus autofluorescence (AF) signal comparable to that using 486 nm excitation light. The detector setting (88) was adjusted to avoid saturation using 486 nm excitation light and remained fixed for all recordings. A laser power of about 0.52 mW (arbitrary laser power of 25) for 642 nm excitation and of about 1.13 mW (arbitrary laser power of 75) for 705 nm excitation resulted in approximately equivalent grey values for the 3 healthy subjects. At 486 nm, the arbitrary laser power of 100 (standard in clinical routine imaging) is equivalent to 0.28 mW.

Supplemental Results 7: Arbitrary units for quantitative analysis of autofluorescence intensity

For a given signal strength (fluorescence yield) the gray value (GV) of the signal is given by the following equation:

$$GV_{total} = GV_{offset} + GV_{signal} \tag{1}$$

GV_{offset} is the signal, which is measured in the dark mode, i.e., when no fluorescence is excited. The Spectralis system measures this value during the resetting period of the slower Y-scanner, when the laser is modulated off and saves it together with other information in the meta-data of the images (personal communication with Dr. Jörg Fischer, Heidelberg Engineering). The GV_{offset} can be read out in the info menu of the Spectralis viewing module.

To analyze the autofluorescence (AF) signal we therefore subtracted the GV_{offset} value from each acquired image and plotted only the remaining part $GV_{signal} = GV_{total} - GV_{offset}$. The gray value GV_{signal} depends on the one hand on system dependent parameters such as the laser power P_0 and the detector sensitivity D, which is adjustable (sensitivity wheel of the Spectralis system). On the other hand, patient related properties such as the density of fluorophores at the ocular fundus, the transmission of the ocular media, and the pupil size determine the signal strength. Finally, user related skills in adjusting the scan pupil of the Spectralis camera onto the entrance pupil of the patient´s eye (lateral alignment, working distance and focus) can impact the measured gray values. The influence of the user can be measured and estimated by repeatability studies (Supplemental Results 8). In the present study, only experienced users acquired images, therefore the user influence is not considered in the following. Of note, time-dependent signal changes due to bleaching of the photopigment within the retina can be neglected for the wavelengths at 642 nm, 705 nm, and 785 nm (Supplemental Results 3).

The goal of the following considerations is to establish an equation, which allows for comparison of AF signals acquired at different detector settings. To compare the AF signals acquired at different power and detector settings, the following equation can be established, if the power and detector settings do not cause any saturation effects of the 8-bit gray value signal.

$$GV_{signal} = P_0 \cdot C_{patient} \cdot S_{det} \tag{2}$$

$C_{patient}$ includes all patient dependent parameters as described above. The detector sensitivity S_{det} depends on the wavelength dependent properties of the avalanche photodiode $S_{APD}(\lambda)$ and on the adjustable detector sensitivity D, which controls two independent gain parameters: the high voltage gain g_{HV} of the avalanche photodiode (APD) as well as the digital gain g_{dig} of the electronic amplification stage, just before the signal voltage is digitized.

$$GV_{signal} = P_0 \cdot C_{patient} \cdot S_{APD}(\lambda) \cdot g_{HV} \cdot g_{dig} \tag{3}$$

For a large range of detector settings D, i.e. in the range between D=52 and D=99, the high voltage gain is constant, whereas the digital gain increases by a constant factor a, when at the Spectralis sensitivity setting D is increased by +1 (personal communication with Dr. Jörg Fischer). In this detector range, equation (3) can be written for a given patient, excitation wavelength and power as:

$$GV_{signal} \propto P_0 \cdot 10^{(D \cdot log(a))} \tag{4}$$

When plotting (for the same patient) the logarithm of the mean gray values in a well-defined area versus detector settings (52 < D < 99), one obtains:

$$\log(GV_{signal}) = const. + log(a) \cdot D \tag{5}$$

The manufacturer of the Spectralis provided us a typical value for a=1.10 resp. log(a)=0.0416 (personal communication with Dr. Jörg Fischer). The parameter a represents the gain increase when the detector setting is increased by one increment. We then plotted the offset-corrected gray value data averaged over a certain image range against the detector setting D for several normal test persons (for 642 nm and 705 nm separately). Afterwards, we plotted the logarithm of the gray value for each data set versus the parameter D and extracted from the linear fit the slope log(a). When calculating the fit parameters only data for D ≤ 90 were used, since for higher amplification e.g. at D=95, first small saturation effects due to the clipping of the averaged gray value distribution at GV=255 was observed. The mean slope log(a)=0.0393 was derived from in-vivo measurements on healthy human volunteers and corresponds to a gain factor of a= 1.0947 per sensitivity step. This is in good agreement with the typical design value provided by the manufacturer Heidelberg Engineering.

This reasonable agreement encouraged us to establish a formula, which allows for calculating from each gray value distribution acquired at a detector setting within the range of 52<D<99 the expected gray value at a detector setting 88, always with the restriction, that the measured gray values are far away from the upper and lower clipping limits:

$$\boldsymbol{GV_{signal}(88) = GV_{signal}(D) \cdot 10^{0.0393 \cdot (88-D)} = GV_{signal}(D) \cdot 1.0947^{(88-D)}}$$

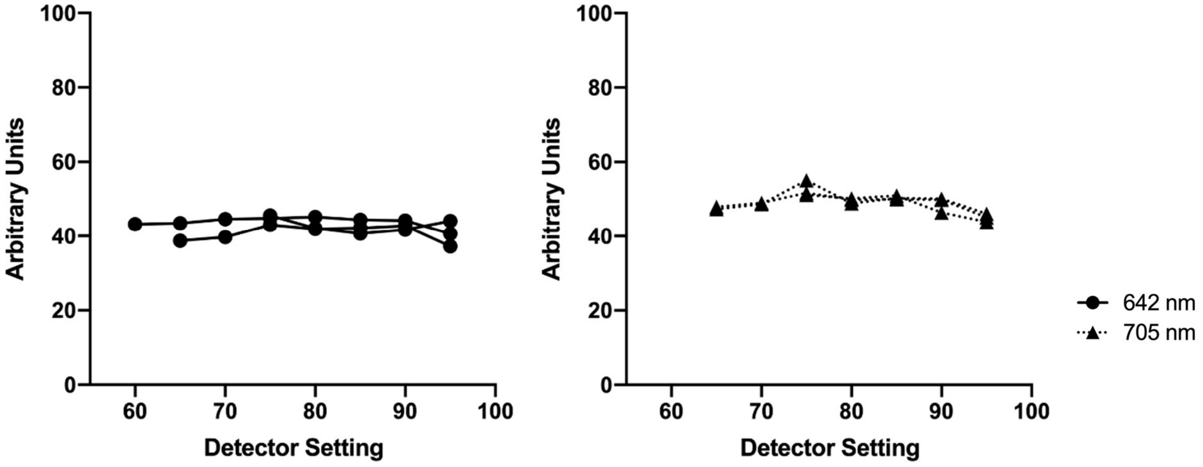

The graphs above illustrate arbitrary units (AU) derived from red excitation fundus autofluorescence (R-AF) images with fixed laser powers and changes in the detector settings of 3 normal eyes. Grey levels were determined by the detector settings and followed the above established equation of $GV_{signal}(88) = GV_{signal}(D) \cdot 10^{0.0393\cdot(88- \ )}$. Here, GV is the measured grey value, D the detector setting used for recording of the image, and 88 is an arbitrarily chosen detector setting that usually results in mid-range grey levels when the standard 486 nm AF imaging is performed in a middle-aged adult without retinal disease. This equation provides arbitrary units (AU) of the AF signal intensity and facilitates quantitative analysis with different detector settings.

Reference:

1. International Electrotechnical Commission. Safety of laser products - Part 1: Equipment classification and requirements. IEC 60825-1:2014.

Supplemental Results 8: Repeatability measurements

To validate the image processing accuracy, repetitive analysis of the same images (acquisition mode: composite) was performed and confirmed the robustness of our pipeline. A repeatability of ±0.5% for the fovea and ±0.5% in the central eight-segments using 642 nm excitation light (n=18 image pairs) and of ±0.8% for the fovea and ±0.8% in the central eight-segments using 705 nm excitation light (n=18 image pairs), indicated only minor position alterations of the grid illustrated in Supplemental Figure 10.

To compare different modalities for image alignment, fundus images were recorded and processed using the composite, mean, and ART (automatic real-time averaging) mode. While the composite and mean mode process images after acquisition, the ART mode is characterized by continuously aligning and averaging frames into one image during acquisition. Qualitative inspection showed no obvious differences between images obtained with these three imaging modalities when detector and laser power settings were in an optimal range. The composite mode was inferior to the mean and ART mode at low detector setting or low laser power, where images are increasingly granular. Quantitative analysis using different detector and laser power settings also demonstrated a strong agreement between the composite, mean, and ART mode. The strongest agreement was found between the composite and mean mode with ±2.2% for the fovea and ±2.8% in the central eight-segments using 642 nm excitation light (n=20 image pairs). Using 705 nm excitation light, agreement was ±9.7% for the fovea and ±6.3% for the central eight-segments (n=18 image pairs), respectively. Agreement between composite and ART mode was ±8.7% for the fovea and ±8.6% in the central eight-segments using 642 nm excitation light (n=12 image pairs). Using 705 nm excitation light, agreement was ±6.1% for the fovea and ±5.9% for the central eight-segments (n=17 image pairs), respectively. Finally, agreement between mean and ART mode was ±5.4% for the fovea and ±4.4% in the central eight-segments using 642 nm excitation light (n=10 image pairs). Using 705 nm excitation light, agreement was ±6.8% for the fovea and ±6.8% for the central eight-segments (n=11 image pairs). However, these and the following repeatability measures indicate that higher variation between 2 measurements would only occur in 5% of occasions (95% confidence interval).

The repeatability within a session as well as between sessions on the same day was investigated on eight subjects using the composite mode. For repeatability testing within-session, successive images pairs (n=1-3 per subject) were acquired within a session (≈2–6 seconds apart) using the same detector setting, laser power, focus, positioning in the chin/headrest, and alignment of the camera. Repeatability was ±5.3% for the fovea and ±4.3% in the central eight-segments using 642 nm excitation light (n=16 image pairs). Using 705 nm excitation light, repeatability was ±6.4% for the fovea and ±7.1% for the central eight-segments (n=17 image pairs), respectively. For image pairs obtained between sessions on the same day (<2 minutes apart, n=1-3 per subject), the same detector and laser power settings were used, but subjects moved away from the instrument and the focus was changed. A slightly higher variability than in the within-session measurements was seen. The repeatability using 642 nm excitation light was ±6.4% for the fovea, ±7.3% for the central eight-segments (n=13 image pairs); using 705 nm excitation light repeatability was ±6% for the fovea, ±7.5% for the central eight-segments (n=19 image pairs), respectively.

Supplemental Results 9: Quantitative analysis of lipofuscin-associated fundus autofluorescence (AF) in a patient with *ABCA4*-related retinopathy (Stargardt disease) and controls

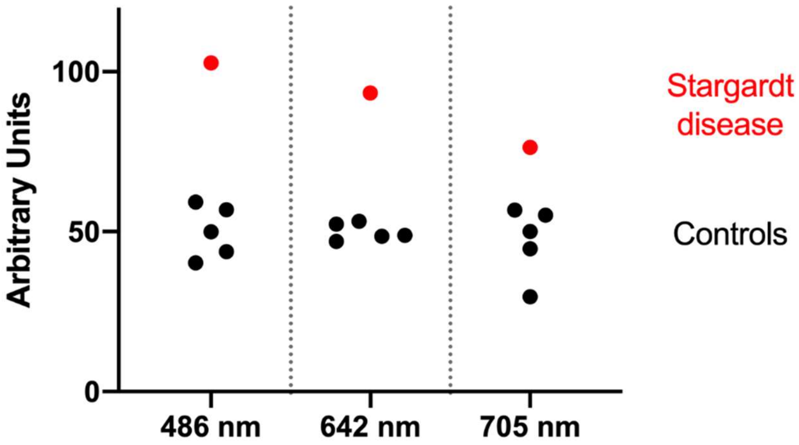

Compared to age-matched healthy controls (black dots), a patient with Stargardt disease (red dots) showed increased fundus autofluorescence (AF) intensities using excitation light of 486 nm, 642 nm, and 705 nm. The relative difference between the controls and the patient was comparable using 486 nm and 642 nm excitation light, whereas the difference was less pronounced at 705 nm excitation light. For visualization, the mean of the quantitative AF measurements of the controls was normalized to the arbitrary unit (AU) of 50. For this quantitative analysis, the central eight-segments as illustrated in Supplemental Methods 2 were used.

Supplemental Methods 1: Laser safety considerations

Autofluorescence (AF) imaging requires consideration of two hazards: (I) the thermal retinal hazard and (II) the photochemical retinal hazard. When excitation wavelengths above 600 nm are used, photochemical risks do not require consideration.[1] Hence, more laser energy can be applied to compensate for potentially lower fluorescence efficiency.

In the following tables, only the long exposure times, which are the most critical case for continuous SLO imaging, are evaluated. The scan pattern was considered as an extended source illumination (table 4 in the referenced standard[1]) with a repetition rate corresponding to the frame rate of the SLO system. For a rigorous and complete laser safety analysis all three rules, listed in the referenced standard[1] in section 4.3.f need to be verified. In the table for photochemical hazard three different time regimes are considered only for the SW-AF laser, whereas in the table for thermal hazard the limits are evaluated for exposure times 100 s < t < 8.3 hours and for different laser wavelengths between 486 nm and 785 nm.

The laser safety of red excitation fundus autofluorescence (R-AF) imaging was compared to short-wavelength AF (SW-AF; excitation light of 486 nm) and near-infrared AF (NIR-AF; excitation light of 785 nm). The ratio of the accessible emission and accessible emission limit (AE/AEL) of R-AF was slightly below the AE/AEL ratio of the NIR-AF laser system (as used in the commercial Spectralis system) due to the lower laser power values. All maximum laser power values were ≈ 8-12 times lower compared to the limits for class 1 laser devices. For the blue laser at 486 nm (SW-AF) the AE/AEL ratio for thermal hazard is less critical due to the significant lower laser power, however, the AE/AEL ratio for photochemical hazard is in a similar range (12%) as the thermal load for the red and NIR laser wavelengths.

<table>
<tr><th colspan="5">Photochemical hazard (for different exposure times)</th></tr>
<tr><th></th><th colspan="3">SW-AF (486 nm)</th><th>R-AF/NIR-AF (642, 705, 785 nm)</th></tr>
<tr><td>Acquisition duration</td><td>Fast acquisition (<100 sec)</td><td>Intermediate acquisition (assumed max. clinical exposure) (e.g., 10 min)</td><td>Max. exposure as used in safety standards (8.3 h)</td><td rowspan="8">excitation wavelengths > 600 nm:

photochemical risks do not require consideration</td></tr>
<tr><td>Time regime in seconds [s]</td><td>t = 10 - 100</td><td>t = 100 - 10^4</td><td>t = $10^4 - 3 \cdot 10^4$</td></tr>
<tr><td>Wavelength dependency:
$C_3 = 10^{0.02\ (\lambda - 450nm)}$</td><td colspan="3">$C_3$ = 5.25</td></tr>
<tr><td>AEL with γ_{ph}
(as defined in standard)</td><td>$3.9 \cdot 10^{-3} \cdot C_3\ [J]$ = 20 mJ

with:
γ_{ph} = 11 mrad</td><td>$3.9 \cdot 10^{-5} \cdot C_3\ [W]$ = 0.204 mW

using:
$\gamma_{ph} = 1.1 \cdot t^{0.5}$ mrad</td><td>$3.9 \cdot 10^{-5} \cdot C_3\ [W]$ = 0.204 mW

using:
γ_{ph} = 110 mrad</td></tr>
<tr><td>laser power (30°· 15°)</td><td colspan="3">280 µW</td></tr>
<tr><td>$\frac{\gamma_{ph}^2}{30° \cdot 15°}$</td><td>$8.8 \cdot 10^{-4}$</td><td>$8.8 \cdot 10^{-6} \cdot t = 0.0053$

(for t=600 s)</td><td>0.088</td></tr>
<tr><td>AE (within γ_{ph})</td><td>$280 \mu W \cdot 100 s \cdot 8.8 \cdot 10^{-4} = 0.025\ \mathrm{mJ}$</td><td>$280 \mu W \cdot t\ 8.8 \cdot 10^{-6} = 1.48\ \mu W$

(for t=600 s)</td><td>$280 \mu W \cdot 0.088 = 0.0246\ \mathrm{mW}$</td></tr>
<tr><td>AE/AEL</td><td>0,12%</td><td>0.0012% · t = 0.72%

(for t=600 s)</td><td>12.1%</td></tr>
</table>

SW-AF short-wavelength fundus autofluorescence using the standard Heidelberg Spectralis BAF (blue autofluorescence mode at 486 nm

R-AF red excitation fundus autofluorescence

C_3 wavelength dependent factor accounting for the fact, that emission at shorter wavelengths consists of photons with higher energy and therefore has higher photo-chemical hazard.

λ wavelength

γ_{ph} limiting measurement aperture of acceptance. For extended light sources, the standard defines a certain angular limitation of the light source, which corresponds to a certain spatial field size on the retina. Only the portion of the emission power, which falls into this limiting acceptance angle range, needs to be considered for determination of the accessible emission. The limiting acceptance angle leads to the fact, that the same emission power of the instrument is less critical, when a large field of view is scanned (wide field imaging) compared to the case where all the emission power is applied within the limited angular field of γ_{ph} x γ_{ph}.

30°x15° angular emission range of the Spectralis in the most critical 15° x 15° imaging mode.

AE accessible emission, i.e. maximum light energy (for short irradiation) or light power (for longer irradiation), which is applied by the instrument to the eye.

AEL accessible emission limit of class 1 laser products[1] which depends on the examination time, the wavelength, and pulse characteristics, i.e. the maximum laser energy resp. laser power, which is corresponding to the referenced standard the safety limit for class 1 laser products.

Retinal thermal hazard (for t > 100s)					
Fluorescence mode	SW-AF (Standard Spectralis)	R-AF		NIR-AF (Study SLO)	NIR-AF (Standard Spectralis)
Wavelength [nm]	486 nm	642 nm	705 nm	785 nm	785 nm
parameter C_4 $C_4 = 10^{0.002(\lambda-700)}$	1	1	1.023	1.48	1.48
parameter C_6	valid for wavelength range 400-1400 nm: 66.7				
parameter T_2	valid for wavelength range 400-1400 nm: 100 s				
Thermal AEL for extended source and long examination time [mW]: $AEL_T = 7 \cdot 10^{-4} \cdot T_2^{-0.25} \cdot C_4 C_6$ (with C_4 =1 for λ< 700 nm)	14.8 mW	14.8 mW	15.1 mW	21.9 mW	21.9 mW
AE [mW] (max. laser power at 30° HR-mode)	0.28 mW	1.3 mW	1.4 mW	2.4 mW	2.8 mW
Laser power causing comparable thermal load as Spectralis ICGA at 2.8 mW	1.89 mW	1.89 mW	1.94 mW	2.8 mW	2.8 mW
AE/AEL for thermal hazard for the AF modes in this study	1.9%	8.8%	9.3%	11.0%	12.8%

C_4 This parameter accounts for the wavelength dependency of thermal damage: For wavelengths below 700 nm this factor becomes 1, for longer wavelengths the parameter C_4 increases with increasing wavelength resulting in higher values for AEL. This factor accounts for the lower absorption and higher penetration depth at longer wavelengths.

C_6 This parameter accounts for the angular subtense and is 66.7 for all modalities used. For point source with angular subtense smaller than 1.5 mrad x 1.5 mrad C_6 is set to C_6=1. For extended light sources, which illuminate a certain retinal area with an angular subtense of α, the parameter C_6 is defined as $C_6 = \alpha_{max}/\alpha_{min}$, with α_{max} as given in table 9 of the referenced standard.

T_2 time parameter as given in table 9 of the referenced standard. This time parameter depends on α_{max} and thus implicitly also on the exposure time t.

HR high resolution scanning mode. The scan patterns of the 15°, 20° and 30° scan fields using the high resolution or high-speed mode are identical to the scan pattern of the commercial Heidelberg Spectralis.

AEL_T accessible emission limit of class 1 laser products[1] which depends on the examination time, the wavelength, and pulse characteristics.

Reference

1 International Electrotechnical Commission. Safety of laser products - Part 1: Equipment classification and requirements. IEC 60825-1:2014.

Supplemental Methods 2: Measurement areas for image analysis

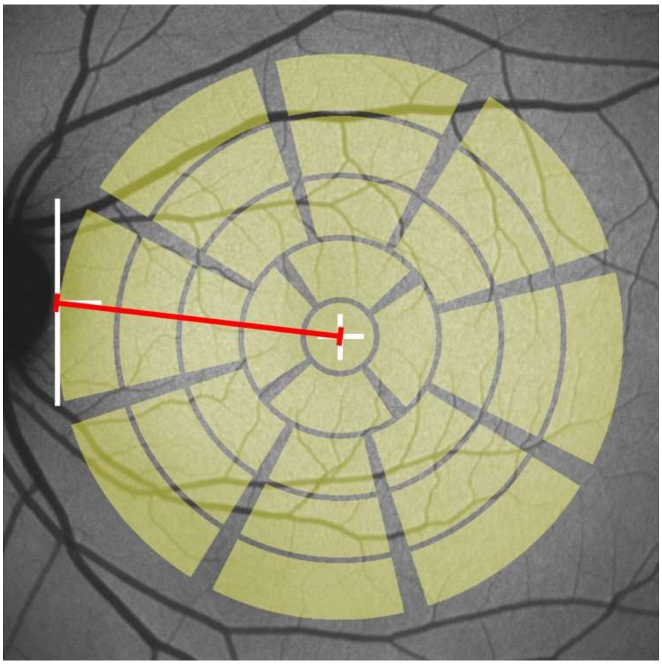

Measurement areas (yellow) were organized in four concentric rings. The outer three rings were divided into eight segments (outer [8-3], middle [8-2], central [8-1]) and the innermost ring was divided into four segments, a circular segment covered the foveal area. All segments were aligned with the horizontal distance (FD, red line) between the **f**oveal center (white cross) and the temporal edge of the **d**isc (white vertical line). The radii expressed in pixels for the outer, middle, central, and innermost ring were 0.90 x FD, 0.68 x FD, 0.46 x FD, and 0.24 x FD. The thickness of all segments was 0.2 x FD. The angles subtended by the outer, middle, and inner segments were 40°, the angles subtended by the innermost segments are 85°. The foveal circular segment had a radius of 0.1 x FD.
